# In Vitro Ecological Response of the Human Gut Microbiome to Bioactive Extracts from Edible Wild Mushrooms

**DOI:** 10.3390/molecules23092128

**Published:** 2018-08-23

**Authors:** Emanuel Vamanu, Florentina Gatea, Ionela Sârbu

**Affiliations:** 1 University of Agronomic Science and Veterinary Medicine, Faculty of Biotechnology, 59 Marasti Blvd, 1 District, 011464 Bucharest, Romania; 2 Centre of Bioanalysis, National Institute for Biological Sciences, 296 Spl. Independentei, 060031 Bucharest, Romania; flori_g_alexia@yahoo.com; 3 ICUB-Research Institute of the University of Bucharest, 36–46 Bd. M. Kogalniceanu, 5th District, 050107 Bucharest, Romania; ionela24avram@yahoo.com

**Keywords:** gallic acid, mushroom, microbiota, *Boletus*, in vitro, three stage culture system

## Abstract

This study presents the effect of two new products based on atomized extracts from edible wild mushrooms (RoBioMush1, RoBioMush2) on the microbiota of three target groups: clinically healthy (NG) individuals, individuals with nutritional disorders (ND), and individuals with cardiovascular diseases (CVD). The microbiota fingerprints were determined by quantitative polymerase chain reaction (qPCR). Modulations in the simulated microbiome were established and correlated with the presence of phenolic compounds released in the in vitro environment (a three-stage culture system GIS2 simulator, www.gissystems.ro). The high metabolizing capacity of NG and CVD correlated positively with the rest of the biological activities expressed in vitro. ND microbiota consumed a wide spectrum of monosaccharides from the products. Xylose was present in large quantities in the descending segment (minimum: 175 μg/mL for ND). The primary conclusion was that the microbiological ecosystem was modulated, as proven by the presence of specific biomarkers (e.g., ammonium levels and fingerprints of short-chain fatty acids–SCFAs), which stimulate the organism’s health status and were correlated with the restoration of a normal microbiota fingerprint.

## 1. Introduction

Edible wild mushrooms and their functional extracts can act as natural healers without presenting any toxicity to humans. In vitro studies demonstrated wild mushrooms’ capacity to improve the microbial fingerprint by stimulating favorable strains of bacteria [1]. The most effective elements from these foods are polyphenolic compounds and the composition of dietary fibers [2]. A large part of phenolic compounds is associated with the presence of these fibers, and they are released in the colon following the fermentative actions of the microbiota [3]. The microbiota fingerprint could be linked to different pathological conditions and a comparison between the chosen target groups is relevant for determining a characteristic profile [4]. The relationship between the fermentative profile and microbiota fingerprint plays a significant role in the mitigation of different pathological effects of microbiota dysfunction, which could promote human well-being [5]. By in vitro modulation of microbiota fingerprint, we could test bioactive molecules that lead to the development of new health therapies [4].

A functional combination of edible wild mushrooms can serve as a potent product with antioxidant activities [6]. The most frequently used species in the market are *Boletus* [7], which are generally used as food components. Truffles, *Armillaria mellea* or *Pleurotus eryngii*, also represent a rich substrate in many bioactive compounds that are widely used in the improvement of health-related disorders caused by oxidative stress [8]. Various species were tested in recent studies to assess their effects on human health and the action of free radicals, particularly when combined with wild mushrooms [2]. These effects were mediated by bioactive compounds, such as gallic acid. This phenolic acid is a compound with different biological activities, as proven by *in vitro* studies on mushrooms [9]. Most of these compounds are not resistant to the fermentative action of microbiota and exert selective action. This observation is related to studies concerning the stability and functional properties of yerba mate [10]. 

The aim of the present study was to determine the in vitro effects of two products based on the extracts of edible wild mushrooms. The human microbiome of three target groups was analyzed. Simulations in the three-stage colonic system (GIS2) established changes in the microbiome fingerprint and antioxidant potential. The presence of specific biomarkers indicated the microbiological modulation. The data were correlated with the presence of phenolic compounds released in the in vitro environment.

## 2. Results

### 2.1. In Vitro Effects of a Microbiome Environment on the Fingerprints of Bioactive Compounds

The comparative analysis of the composition in monosaccharides and phenolic compounds of the two atomized extracts before (Table 1) and after administration (Table 2) is presented. Thus, significant amounts of umbelliferone and cinnamic acid (*P* < 0.05) were identified in the ND group. Statistically differences were determined for the same target group after the addition of the two products. The modulating action was more pronounced for the CVD group, where only traces of sinapic acid (86.67 μg/mL) were recorded; this value was characteristic for the terminal segment of the colon. The gradual reestablishment of the microbiome fingerprint determined that the phenolic fraction had a profile that was similar to that of the NG group, which completely metabolized the same bioactive compounds.

The carbohydrate content followed a characteristic pattern (Table 1 and Table 2A,B). The balanced fingerprint of the NG group was translated by a determined minimum residual number of compounds that showed greater resistance to fermentative action (xylose). RoBioMush 2 was characterized by the presence of galacturonic acid. The ND microbiota had the lowest consumption and determined a wide spectrum of monosaccharides in the GIS2 system. Also, xylose may be considered characteristic following RoBioMush administration, as it is present in large quantities in the terminal segment of the colon (min 175 μg/mL for ND).

### 2.2. In Vitro Effects of the Microbiome Environment on Antioxidant Capacity

**Figure 1:**
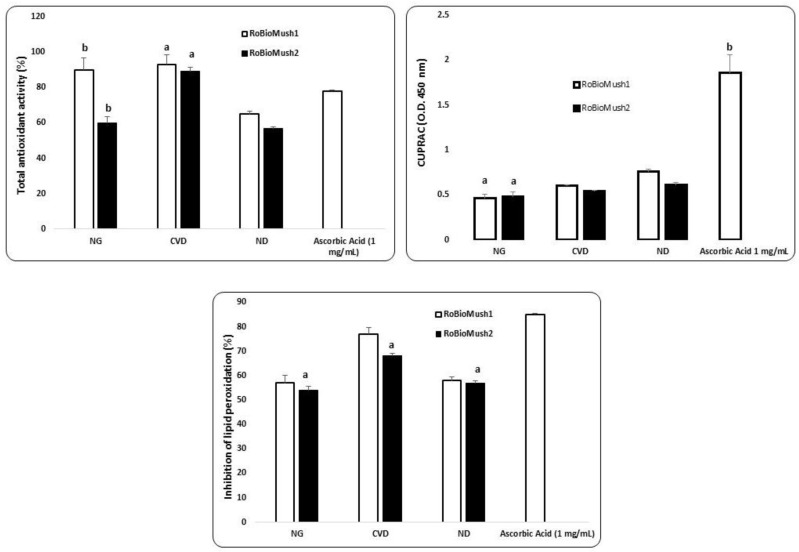
*In vitro* antioxidant activity. Different letters mean significant statistical differences (*P* ≤ 0.05).

The average antioxidant capacity recorded following the in vitro simulation is presented in Figure 1. The fermentative action of the colon microbiota had an effect whereby it increased the antioxidant capacity from one segment to another for RoBioMush2. The exception was the ND group in terms of its reduction power values (Figure 1). High antioxidant potential for the CVD group was highlighted, together with the presence of a large number of phenolic compounds and a decrease in the number of negative potential strains (for example, *Escherichia coli*).

Lipid peroxidation inhibition presented a particular pattern for the CVD group (Figure 1), where a 26% ± 0.08% increase when compared to the NG group was observed. The high presence of phenolic fraction (Table 1 and Table 2) was interpreted as source of antioxidant potential, express as inhibition of lipid peroxidation in the case of RoBioMush1 for CVD. In the present study, the protection at lipid peroxidation was gradually increase in the case of CVD. In comparison, ND group presented a similar capacity of protection at lipid peroxidation with NG (Figure 1).

### 2.3. In Vitro Effects of the Microbiome Environment on Metabolic Activity

The administration of the two atomized products had a strong effect of normalizing the quantity of organic acids produced for the CVD group, but it also demonstrated a reduction in the level of synthesized ammonium. Metabolism intensification was also observed by the level of SCFAs, which showed maximum values of 8.38 mg/mL for the same group (Table 2C), which occurred simultaneously to the presence of a high number of *Lactobacillus* strains. In contrast, the administration of inulin as a control did not enhance metabolism, even if it stimulated the multiplication of favorable strains (especially for the ND group; e.g., butyric acid 0.88 ± 0.02 mg/mL). Thus, it can be assumed that the ecological response of the microbiota was inversely proportional to inulin administration without a tendency of intestinal persistence. The SCFA level, considered to be a biomarker of the microbiome response, was similar to that of the untreated microbiotas (e.g., butyric acid 2.05 ± 0.07 mg/mL). The high level of lactic acid production for NG was also supported by an increase in butyric acid and acetic acid (especially following the administration of RoBioMush1), which coincided with a >20% increase in the quantity of ammonium.

### 2.4. Quantifying the Number of Microbial Cells from the Microbiota

**Figure 2:**
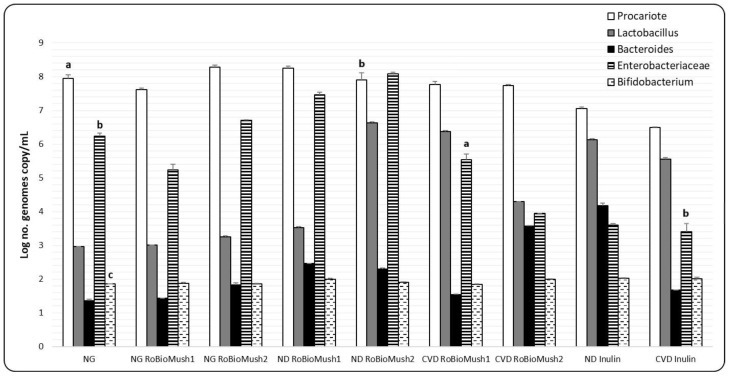
Number of microorganisms in the analyzed samples. Different letters mean significant statistical differences (*P* ≤ 0.05).

These new products differentially influenced the microbiota taken from patients with ND and CVD. The RoBioMush1 product caused an increase in the number of enterobacteria in the microbiota taken from ND patients by almost two decimal places, as well as the increase in the number of lactobacilli in the case of the microbiota taken from patients with CVD by almost three decimal places (Figure 2). The microbiota taken from patients with ND, RoBioMush2, induces a three-decimal-place increase in the number of lactobacilli, and a one-decimal-place increase in the number of enterobacteria; furthermore, the *Bacteroides* group was also slightly stimulated by the presence of this product. Microbiota from CVD patients was affected differently by the RoBioMush2 product, which induced a one-decimal-place growth in the number of lactobacilli and a two-decimal-place growth in the number of *Bacteroides*, while there was an enterobacteria decrease of three decimal places compared with the control. Inulin causes an increase in the number of lactobacilli and a decrease in the number of enterobacteria (Figure 2).

## 3. Discussion

The research highlighted the microbial response of the three target groups microbiota to the addition of the two functional products. The selection of the target groups was based on the significance of microbiota fingerprint modulation that may determine benefits in alleviation of two pathologies with high incidence. As new functional products are formulated and validated, the possibilities to improve treatment for these target groups expand [11]. 

The modulating capacity of the two products selectively stimulated the favorable components. GIS2, a three-stage culture system, was efficient and capable of demonstrating the ability of the colon’s bacterial community to selectively ferment certain bioactive components from the tested products. Thus, for the NG microbiota, there was a rapid increase in in vitro antioxidant potential immediately after the addition of RoBioMush1, which is a characteristic behavior of the ascending segment of the colon. After 4 h of fermentation from ascending to transverse segment passage, the pH stabilized and the number of strains of the *Lactobacillus* genus, for example, remained constant. The other target groups showed a different biochemical and microbiological behavior, caused by the microbiota fingerprint differences. The stabilization period was reached only when passing to the descending segment. With the significant variation of the antioxidant potential (*P* < 0.05) from the first two simulated segments, stabilization of the microbiological fingerprint from the transverse segment was simultaneously recorded. This characteristic microbiological behavior was inversely proportional (*R*^2^ < −0.5) to the total amount of phenolic compounds because the profile induced or suppressed metabolic activity of the population of specific gut bacteria. A characteristic profile was observed for each target group throughout the entire in vitro transit through the human colon [12]. The umbelliferone and cinnamic acid determined the characteristic odor of RoBioMush1 containing *T. melanosporum*. 

Changes between certain groups of microorganisms from microbiota could not be associated with a significant degradation of certain compounds, such as isoquercetin, which has antiproliferative capabilities and can thus be used to regulate other essential physiological functions. In contrast, the resistance previously identified for the ND group [9] demonstrated the presence of numerous and diverse unused bioactive compounds (especially flavonoids) that can be eliminated naturally. A significant reduction in the number of phenolic compounds that resulted following the in vitro simulations demonstrated that the bacteria in the human colon determine a bioconversion that positively modulates the microbiome fingerprint [13]. The high metabolizing of bioactive compounds capacity of the NG and CVD groups correlated positively with the remaining biological activities expressed in vitro.

The antioxidant potential registered after products addition was confirmed by previous studies and was primarily correlated with the number of phenolic compounds (*R*^2^ > 0.7) [14]. Considering one of the purposes of this study, it could be argued that the antioxidant potential demonstrated in vitro was similar to that expressed by the hydroalcoholic extracts from the fruiting body of the same species [15]. This observation also applied to the mycelium and the fruiting body of some species such as *Agaricus bisporus* and *Pleurotus ostreatus* [16]. These innovative results demonstrated that, compared to lyophilizates [17], atomized products did not present the significant component losses (e.g., isoquercetin or chlorogenic acid) responsible for expressing biological activity.

Similar to what was reported in previous studies [18], the main microbiological component that varied was represented by the favorable strains of the microbiota, *Lactobacillus* spp. and *Bifidobacterium* spp. The variation was recorded, especially for RoBioMush1, in the first two segments and was sustained by the different levels of antioxidant activity in the ascending segment. Instead, RoBioMush2 showed a variation in the phenolic component and in the amount of ammonium among the ND group. The CVD group exhibited a linear increase in the main components, including a constant evolution in the accumulation of ammonium and SCFAs (Table 2C). 

In general, it may be considered that, depending on the target group analyzed, RoBioMush1 and RoBioMush2 had a positive effect on the intestinal microbiome, both by modulation of the microbiota fingerprint and by their metabolic activity. The ratio between different metabolic products (e.g., organic acids) and the number of favorable strains determined after the ingestion of these atomized species directly proved the ecological response. Microbiome modulation was much stronger after RoBioMush1 administration to the CVD, as it coordinated both the microbiological response to human colon passage and the reduction of oxidative stress occurring in the case of the modified fingerprint.

Due to their similar levels of acetic acid and butyric acid, the formulated products (especially RoBioMush2) may have an impact on decreasing cholesterol synthesis. This behavior was correlated with the reduction of lipid peroxidation and translated into high anti-inflammatory protection, according to previous studies [18]. In the case of the ND group, the SCFA profile also demonstrated an increase in propionic acid compared to the CVD group or to inulin addition as a control (0.11 ± 0.02 mg/mL). Despite the positive metabolic response to the SCFA level, the increase in the ammonium level (over 20%, at a maximum level of 50 mmol/L) explained the negative response in the change of the microbiome fingerprint for the target groups.

The modulation of the ecological response from the microbiota level was thought to largely result from to the non-extractable polyphenolic component, which is the fraction that predominantly controls the microbial fingerprint of the colon. Due to its selective action, the polyphenolic fraction may be considered to have prebiotic action, although the direct effect on the favorable component was not a stimulation of cellular multiplication. The “prebiotic” action was manifested by its use as a growth factor (for *Bacteroides*, *Enterococcus*, *Bifidobacterium*, and *Lactobacillus*), and an antimicrobial effect on the undesirable fraction of the microbiota was also registered. A significant aspect was the persistence of these bioactive compounds throughout the simulated colon. In addition to high stability, it was found that modulating action caused an effective response to oxidative stress by increasing antioxidant potential values, especially in the descending segment (Figure 1). Modulation of the microbiological ecosystem resulted in a positive change in colonic metabolism of the target groups, a reduction in ammonium level, and a distinct fingerprint of SCFAs. According to current studies, the presence of these specific biomarkers stimulates the health status and restoration of a normal microbiota [19].

After the addition of RoBioMush products, a pattern of monosaccharides was identified. Depending on the quantity recorded, we may consider that these monosaccharides acted by modulating the biological response, together with the presence of the phenolic fraction (Table 2). The differences were obvious, according to the presented data, as the ND group had a lower use of carbon sources compared to the other two target groups. The modulation of the CVD group through the RoBioMush1 product was a normalization of bioactive compound consumption. The two product formulations determined the presence of significant amounts of arabinose, glucose, galactose, and glucuronic acid at the level of the colon’s terminal segment (Table 2). It is important to note the catabolic profile manifested by RoBioMush1 when a residual amount of xylose and arabinose deficiency were recorded. These biochemical characteristics were correlated with the normalization of the microbial fingerprint and a correction of the CVD microbiome.

The correlation between the metabolism of the major bioactive components from the two products and the degree of resistance of the microbiome fingerprint represents a significant research direction that involves improvements in lifestyle and health status. Although the samples from each segment presented significant differences (Table 1 and Table 2), the biological action was directly proportional to the concentration of the main metabolites, and in accordance with what was reported in previous studies [20]. These differences are due to the sample analysis and methods adapted to each study. In a previous study [9], gallic acid, a biomarker of the use of dried mushrooms in microbiome modulation, did not show stability after the fermentative action from the simulated colon. The introduction of the two new types of mushroom-based products, along with the microbiota pattern of healthy individuals showed the major changes in the groups of microorganisms analyzed [21].

## 4. Materials and Methods

### 4.1. Standards and Reagents

All chemicals and reagents were purchased from Sigma Aldrich GmbH (Steinheim, Germany). All other unlabeled chemicals and reagents were of analytical grade.

### 4.2. Obtaining Atomized Extracts

The atomized extracts were realized in the following phases, in the Pharmaceutical Biotechnologies lab. For RoBioMush1 (*Agaricus bisporus*, *Chantharellus cibarius*, *Pleurotus ostreatus*, *Boletus* sp., *Craterellus cornucopioides*, and *Armillaria mellea*) and RoBioMush2 (*Boletus* sp., *Pleurotus eryngii*, *Armillaria mellea*, and *Tuber melanosporum*), the mushrooms were harvested, sorted, washed, and dried in air flow to a constant weight. The criteria used for establish every ratio were the disponibility of the materials and biological activity of species harvested from Romanian forests [15]. The mushroom mixture was dosed at a ratio of 1:1:1:1:1:1 (RoBioMush1) and 30:7:2:1 (RoBioMush2), then ground [22]. It was mixed with the extraction enzyme (Viscozyme L; Novozymes, Copenhagen, Denmark) at a rate of 3 mL/kg and subjected to the extraction process in the turbo-extractor (conceptualized and manufactured by Hypericum Impex SRL—http://www.hypericum-plant.ro/) for 3 h at 40 °C. Extract 1 was obtained by pressing the mixture with a drum press. The mushroom residues were introduced into a steam infuser at 120 °C, and Extract 2 was obtained in the same manner as Extract 1. Extracts 1 and 2 were mechanically mixed and the solution was decanted. The products were filtered through high-porosity mechanical filters (made from an austenitic stainless-steel sheet of 0.5–1 mm in diameter) and then introduced into the atomizer. The resulting liquid was mechanically mixed with 70 g of maltodextrin (17% dextrose index) until total homogenization was achieved. The resulting mixture was atomized (Spray-Dry; Niro A/S, Soeborg, Denmark) and the resulting powder was collected [23]. With a capsule-dosing machine, enteric-coated (gastro-resistant) capsules with an average weight of 240 mg/capsule [22] were made.

### 4.3. In Vitro Microbiome Modulation

All the in vitro tests were performed in a three-stage culture system GIS2 simulator (http://gissystems.ro/gis-technology/). The system of in vitro colonic simulation consisted of three Duran bottles (500 mL capacity) made of borosilicate glass with a removable screw cap. The operation principle of the GIS2 system for each colon segment has been described in a previous study, and it was used a continuous fermentation process [24,25]. 

Reconstitution of the target groups’ microbiome (NG, ND—type 2 diabetes and CVD—dyslipidemia groups) was performed after a mean interval of 7–10 days and this process followed the previously described protocol [25]. The volunteers (three for NG, five persons each ND and CVD) were from both sexes, ages between 40 (CVD) and 70 (CVD & (ND). These individuals had not been treated with antibiotics or any other interfering drugs over the past 6 months, as these agents may alter the microbiome fingerprint. The samples (faeces) were handled in accordance with the ethical guidelines of UASVM Bucharest (ColHumB Registration number: 1418/23.11.2017; www.colhumb.com) and analyzed individually. Samples were collected in 10% glycerol and stored at −15 °C until use. Following the removal of large particles, the microbiota was reconstituted in peptone water [9].

The sampling was realized at the level of each colon segment of the simulation process, in the case of each product addition. To test the effect of the atomized extracts on the evolution of microbial communities, three identical series of treatments were performed by adding one dose (capsule) every 12 h in the in vitro simulator. For an effective delivery of the products in the colon, enteric coated capsules were used (size 0; BSC, Wenzhou, China) instead of adding directly the extract. The capsules were directly added in the simulated environment under sterile conditions. At the end of the simulation, each sample was centrifuged (4000× *g*, 15 min, Hettich Universal 320, Hettich GmbH & Co., Kirchlengern, Germany) and the sediment (microbial fingerprint) was kept in glycerol 20% for qPCR analysis. Supernatant was stored in the refrigerator (at −15 °C). 

### 4.4. Quantification of Short-Chain Organic Acids by Capillary Zone Electrophoresis (CZE)

CZE was used for the quantitative and qualitative analysis of the SCFAs. Bacterial cultures were centrifuged at 5000× *g* for 10 min. The supernatants resulted were collected, filtered on 0.2-μm membranes (Millipore, Bedford, MA, USA), degassed and injected into the instrument.

An Agilent CE instrument (ChemStation software) with a diode array detector (DAD) was used to partition the analytes [14]. Separation was achieved on a standard bare fused silica capillary (Agilent Technologies, Santa Clara, CA, USA) with an internal diameter of 50 μm and an effective length of 72 cm. Prior to each use, the capillary was washed successively with basic solutions of: 1 M NaOH for 10 min; 0.1 M NaOH for 10 min; ultra-pure water for 10 min; and migration buffer for 20 min. Between migrations, the capillary was washed with 0.1 M of NaOH for 1 min, H_2_O for 1 min, and a background electrolyte (BGE) for 2 min. The migration buffer was refreshed after three consecutive runs. The sample injection was done in hydrodynamic mode (3500 mbar/12 s) and the capillary was maintained at a constant temperature of 25 °C.

The separation of short-chain organic acids by CZE required a method that employed direct ultraviolet (UV) detection and reverse polarity. This technique was based on the modified Galli and Barbas method [26]. The applied voltage was −25 kV and the best UV detection was achieved at 200 nm. The BGE was composed of 0.5 M H_3_PO_4_, 0.5 mM of CTAB (pH adjusted with NaOH at 6.24), and 15% vol. of methanol as an organic modifier; the buffer was filtered on 0.2 μm membranes (Millipore PTFE, Bedford, MA, USA) and degassed prior to use. The migration order was: acetic, propionic, lactic, and butyric acids. The amount of ammonia was determined by an Ammonium Quantofix kit (Macherey-Nagel GmbH & Co. KG, Duren, Germany) [9].

### 4.5. Quantification of Polyphenols and Carbohydrates

#### 4.5.1. Polyphenol Analysis by CZE

Polyphenol extraction from the two products RoBioMush1 and RoBioMush2 was performed by sonication (the flask was immersed in ice to avoid overheating), over 1 h, with ethanol (70 vol%) in 1:10 ratio (g mL^−1^). Next, the extracts were centrifuged for 15 min at 5000 min^−1^ and the supernatants were collected, concentrated till dryness in a TurboVap and redissolved in 1 mL of methanol. Before the injection the polyphenols extracts were filtered (0.2 μm Millipore PTFE) and degassed. 

Polyphenol separation and quantification were performed by CZE using a DAD-Agilent system. Phenolic acids and flavonoids were separated on a fused silica capillary with an internal diameter of 50 μm and an effective length of 72 cm. As a migration electrolyte (BGE), the 32.5 mM tetraborate buffer was used with 0.9 mM sodium dodecyl sulfate (SDS) adjusted to a pH of 9.025 with 1 M HCl. The method employed a 30 kV voltage, a constant temperature of 30 °C, and direct UV absorption at 280 nm [27].

#### 4.5.2. Carbohydrate Analysis by CZE

The dry material of the two products RoBioMush1 and RoBioMush2 (10 g) were immersed in 100 mL deionized water for 10 h at 4 °C, and then was heated at 80 °C for 1.5 h. The resulting extract was filtered through paper and the solid material was subjected to extraction in the same way again. Next, the extraction solutions were combined, concentrated, and precipitated by adding ethanol to a final concentration of 95% *v*/*v*. The mixture was left overnight (10 h) at 4 °C. The precipitate was collected after centrifugation (6000 rpm, 10 min) and reconstituted with 50 mL deionized water, and completely deproteinated by several rinses with Sevag solution (chloroform/butyl alcohol *v*/*v* 4:1). By adding ethanol to afinal concentration of 95% the supernatant was precipitated, centrifuged, and subsequently dried at 37 °C.

Polysaccharide extract (20 mg) was dissolved in 2 mL of 1.5 M sulfuric acid solution. Polysaccharide hydrolysis was completed at 100 °C for 5 h, and then the samples were cooled and neutralized with1 mL of 6.0 M NaOH solution [28]. Monosaccharide derivatization with1-phenyl-3-methyl-5-pyrazolone (PMP) was attained by adding 200 μL of mixed working standard solution (or the hydrolyzed polysaccharide sample), 100 μL of 0.5 M PMP methanol solution, and 100 μL of 0.3 M NaOH to each test tube. The mixture was left to react for 30 min at 70 °C; then, it was cooled to room temperature and neutralized with 100 μL of 0.3 M HCl solution. Excess derivatization reagent was removed with 10 mL of chloroform and the upper phase was collected and filtered through a 0.45 μm membrane.

The separation of the PMP derivatized monosaccharides was obtained using the same Agilent CE system, capillary, preconditioning, and postconditioning conditions. The selected method was based on the methods of Chen et al. with some changes [19]. The running buffer consisted of 90 mM of boric acid adjusted to a pH of 9.975 with 1 M NaOH solution. The applied voltage was 30 kV, the temperature was kept constant at 30 °C, and detection was fixed to 245 nm [29].

### 4.6. Determination of In Vitro Antioxidant Activity

Two different methods were used to evaluate the antioxidant potential following in vitro simulation: total antioxidant activity (TAA) [23] and cupric ion reducing power (CUPRAC) [30]. Lipid peroxidation was determined by using egg yolk, rich in phospholipids, triacylglycerols and proteins [31,32]. Ascorbic acid (1 mg/mL) was used as a positive control [13].

### 4.7. Microbial Fingerprint Analysis

The microbial DNA was extracted from 1 mL of the culture using the PureLink Microbiome DNA Purification Kit (Thermo Fisher Scientific, Waltham, MA, USA). The concentration and purity of DNA was measured using a NanoVue Plus spectrophotometer (GE, Boston, MA, USA).

qPCR analysis was carried out on a 7900 real-time PCR machine (Applied Biosystems, Foster City, CA, USA) using the Power SYBR Green PCR Master Mix (Applied Biosystems), where 40 ng of the DNA template was introduced in each reaction.

To achieve bacterial quantification, standard curves were developed using serial dilutions of a known DNA concentration corresponding to *Escherichia coli* (American Type Culture Collection [ATCC] 10536), *L. plantarum* (ATCC 8014), *B. breve* (ATCC 15700), and *B. fragilis* (DSM 2151). A bacterial universal primer pair was used to determine the bacterial load from each sample [33]. All samples were run in triplicate.

### 4.8. Statistical Analysis

All investigated parameters were assessed in triplicate, and the results were expressed as the mean ± standard deviation (SD) values of three observations. The mean and SD values were calculated with the Excel program from Microsoft Office 2016 (Microsoft Corporation, Redmond, WA, USA). The significance level was set at *P* ≤ 0.05, and the normal distribution of the variables was used. The IBM SPSS Statistics software package (IBM Corporation, Armonk, NY, USA) was used to analyze and correlate the experimental data.

## 5. Conclusions

The administration of RoBioMush products resulted in the maintenance of homeostasis in the simulated colon. The innovative results support the idea that mushroom consumption can yield functional products due to the effects observed on the tested target groups, especially among the CVD group. The ecological response following product administration was related to the modulation of CVD microbiota, even if the multiplication phase of the favorable strains coincided with an increase in the lactic acid level. The ecological and metabolic response of the microbiome to RoBioMush administration had a direct effect on the phenolic fraction and consumption of prebiotic precursor monosaccharides. These components acted as mediators of the ecological response, with a partial modulating effect for the ND group.

## Figures and Tables

**Table 1:** The main bioactive compounds of the two functional products.

Product (Atomized Formula)	Glucides (g/g Atomized Samples)	Phenolic Acids (µg/g Atomized Samples)	Flavonoids (µg/g Atomized Samples)
RoBioMush1	Xylose 0.033 ± 0.001 ^a^ Arabinose 0.041 ± 0.008 ^b^ Glucose 0.192 ± 0.018 ^c^ Rhamnose 0.115 ± 0.017 ^c^ Mannose 0.007 ± 0.000 Galactose 0.016 ± 0.001 ^a^	Cinnamic acid 1.84 ± 0.03 ^a^ Chlorogenic acid 2.41 ± 0.02 ^a^ Sinapic acid 0.89 ± 0.00 Ferulic acid 0.2 ± 0.00 Coumaric acid 0.08 ± 0.00	Rutin 11.7 ± 0.62 ^b^ Naringenin 0.08 ± 0.00 Luteolin 0.58 ± 0.00 Isoquercitrin 3.6 ± 0.01 ^a^ Umbelliferone 0.65 ± 0.03 ^a^
RoBioMush2	Xylose 0.04 ± 0.002 ^a^ Arabinose 0.033 ± 0.002 ^a^ Glucose 0.239 ± 0.008 ^b^ Rhamnose 0.138 ± 0.001 ^a^ Mannose 0.018 ± 0.000 Galactose 0.026 ± 0.001 ^a^	Cinnamic acid 1.45 ± 0.03 ^a^ Chlorogenic acid 1.36 ± 0.04 ^a^	Rutin 17.6 ± 0.45 ^a^ Naringenin 0.41 ± 0.00 Luteolin 0.55 ± 0.00 Isoquercitrin 2.11 ± 0.02 ^a^ Umbelliferone 2.04 ± 0.01 ^a^

Different letters mean significant statistical differences (*P* ≤ 0.05).

**Table 2:** The average amount of monosaccharides (μg/mL), the phenolic fraction (μg/mL) and organic acids (mg/mL) following simulation in the three-stage culture system.

Bioactive Compounds (µg/mL)	NG * RoBioMush1	NG RoBioMush2	ND * RoBioMush1	ND RoBioMush2	CVD * RoBioMush1	CVD RoBioMush2
**(A)** **Monosaccharides after in vitro simulation**						
Xylose	250.70 ± 6.46 ^d^	-	174.99 ± 2.76 ^b^	172.38 ± 0.92	246.79 ± 4.62 ^b^	-
Arabinose	-	-	127.93 ± 4.73 ^b^	105.04 ± 3.94 ^a^	-	-
Glucose	34.76 ± 1.07 ^c^	-	133.12 ± 3.21	70.32 ± 2.14 ^b^	-	168.13 ± 3.16
Rhamnose	-	-	237.76 ± 2.40 ^a^	-	-	-
Mannose	-	-	224.33 ± 6.50	83.14 ± 0.93	-	13.53 ± 3.70 ^c^
Galactose	81.66 ± 1.94 ^c^	89 ± 1.05 ^c^	230.69 ± 3.89 ^c^	396.68 ± 5.19 ^c^	2.33 ± 0.00	126.14 ± 2.59
Galacturonic acid	-	233.83 ± 5.90 ^d^	-	75.74 ± 2.21	-	76.26 ± 4.42 ^b^
Glucuronic acid	-	-	-	-	-	122.06 ± 2.91 ^a^
**(B)** **The phenolic fraction after in vitro simulation**						
Rutin	-	-	12.88 ± 0.49 ^a^	-	-	-
Naringenin	-	5.46 ± 0.11 ^a^	-	-	-	-
Isoquercitrin	-	83.44 ± 1.36 ^b^	2.29 ± 0.37	-	-	-
Umbelliferone	-	-	4.90 ± 0.61 ^b^	32.23 ± 1.06 ^a^	-	-
Cinnamic acid	-	3.0 ± 0.11 ^a^	-	5.3 ± 0.37 ^b^	-	-
Chlorogenic acid	-	-	-	2.99 ± 1.07 ^a^	-	
Sinapic acid	-	-	-	-	-	86.67 ± 1.64 ^a^
Kaempferol	-	-	-	-	-	-
Luteolin	-	-	-	-	-	1.11 ± 0.00
Coumaric acid	-	-	-	1.57 ± 0.20	-	-
**(C)** **Organic acids**						
Acetic acid	0.48 ± 0.01	1.09 ± 0.00	1.56 ± 0.01	2.67 ± 0.11 ^a^	1.57 ± 0.01	0.55 ± 0.03
Propionic acid	-	0.17 ± 0.01	1,07 ± 0.00	0.73 ± 0.01	0.15 ± 0.00	0.07 ± 0.00
Lactic acid	2.78 ± 0.01	1.39 ± 0.12 ^a^	16.86 ± 2.11 ^b^	23.10 ± 2.69 ^a^	5.35 ± 0.02	7.14 ± 0.45 ^a^
Butyric acid	2.09 ± 0.18 ^a^	0.55 ± 0.03	2.22 ± 0.07	2.56 ± 0.08	1.46 ± 0.03	1.03 ± 0.10 ^b^

* NG—clinically healthy individuals, ND—individuals with nutritional disorders, CVD—individuals with cardiovascular disease; Different letters mean significant statistical differences (*P* ≤ 0.05).
